# Sedentary lifestyle and body composition in type 2 diabetes

**DOI:** 10.1186/s13098-021-00778-6

**Published:** 2022-01-15

**Authors:** Dan-dan Li, Yang Yang, Zi-yi Gao, Li-hua Zhao, Xue Yang, Feng Xu, Chao Yu, Xiu-lin Zhang, Xue-qin Wang, Li-hua Wang, Jian-bin Su

**Affiliations:** 1 Department of Nursing, Affiliated Hospital 2 of Nantong University, and First People’s Hospital of Nantong City, No. 6 Haierxiang North Road, Nantong, 226001 China; 2 Department of Nuclear Medicine, Affiliated Hospital 2 of Nantong University, and First People’s Hospital of Nantong City, No. 6 Haierxiang North Road, Nantong, 226001 China; 3 Department of Endocrinology, Affiliated Hospital 2 of Nantong University, and First People’s Hospital of Nantong City, No. 6 Haierxiang North Road, Nantong, 226001 China; 4 Department of Clinical Laboratory, Affiliated Hospital 2 of Nantong University, and First People’s Hospital of Nantong City, No. 6 Haierxiang North Road, Nantong, 226001 China

**Keywords:** Sedentary lifestyle, Fat distribution, Skeletal muscle mass, Bone mineral density

## Abstract

**Background:**

Body composition alterations may participate in the pathophysiological processes of type 2 diabetes (T2D). A sedentary lifestyle may be responsible for alterations of body composition and adverse consequences, but on which body composition of patients with T2D and to what extent the sedentary lifestyle has an effect have been poorly investigated.

**Methods:**

We recruited 402 patients with T2D for this cross-sectional study. All patients received questionnaires to evaluate sedentary time and were further divided into three subgroups: low sedentary time (LST, < 4 h, n = 109), middle sedentary time (MST, 4–8 h, n = 129) and high sedentary time (HST, > 8 h, n = 164). Each patient underwent a dual energy X-ray absorptiometry (DXA) scan to detect body composition, which included body fat percentage (B-FAT), trunk fat percentage (T-FAT), appendicular skeletal muscle index (ASMI), lumbar spine bone mineral density (BMD) (LS-BMD), femoral neck BMD (FN-BMD), hip BMD (H-BMD) and total BMD (T-BMD). Other relevant clinical data were also collected.

**Results:**

With increasing sedentary time (from the LST to HST group), B-FAT and T-FAT were notably increased, while ASMI, LS-BMD, FN-BMD, H-BMD and T-BMD were decreased (*p* for trend < 0.01). After adjustment for other relevant clinical factors and with the LST group as the reference, the adjusted mean changes [B (95% CI)] in B-FAT, T-FAT, ASMI, LS-BMD, FN-BMD, H-BMD and T-BMD in the HST group were 2.011(1.014 to 3.008)%, 1.951(0.705 to 3.197)%, − 0.377(− 0.531 to − 0.223) kg/m^2^, − 0.083(− 0.124 to − 0.042) g/cm^2^, − 0.051(− 0.079 to − 0.024) g/cm^2^, − 0.059(− 0.087 to − 0.031) g/cm^2^ and − 0.060(− 0.088 to − 0.033) g/cm^2^, *p* < 0.01, respectively.

**Conclusions:**

A sedentary lifestyle may independently account for increases in trunk and body fat percentage and decreases in appendicular skeletal muscle mass and BMD of the lumbar spine, femoral neck, hip and total body in patients with T2D.

## Introduction

Type 2 diabetes (T2D) is a chronic metabolic disease characterized by sustained hyperglycaemia and is a leading cause of morbidity and mortality [1]. Patients with T2D present with numerous pathophysiological disorders, including alterations in body composition. The characteristics of body composition in T2D, including abnormalities in fat distribution, increases in total fat mass, and decreases in muscle mass or bone mineral density (BMD) [2–4], might lead to a wide spectrum of adverse health outcomes, such as cardiovascular diseases (CVDs) [5], sarcopenia and osteoporosis [6–8]. At present, ongoing global research efforts are trying to identify intrinsic and external risk factors for alterations in body composition, which can help guide the development of appropriate therapeutic regimens to modify these pathophysiological disorders and the subsequent prognosis of diabetes.

With the rapid development of China's information society, physical work has become increasingly less common, followed by a sharp increase in the number of sedentary people. Sedentary behaviour refers to any low energy consumption behaviour when a person is awake, including sitting, leaning, or lying down. Studies have shown that sedentary behaviour is inseparable from all-cause mortality, cardiovascular disease mortality and tumour mortality, as well as the incidence of cardiovascular disease, tumours and T2D [9–12]. A survey shows that in the awake state, the sedentary time accounts for at least 50% of total lifetime in T2D patients [10]. Long-term sedentary behaviour is also closely related to the prevalence of human bone and muscle-related diseases [13], leading to an increase in the proportion of lumbar disc disease and a decrease in BMD [14–16]. There is a negative correlation between mineral content [17], the ability of the spinal muscles to maintain maximum output, and muscle strength [18]. A sedentary lifestyle may be responsible for altered body composition and adverse consequences, but on which body composition of patients with T2D and to what extent the sedentary lifestyle has an effect have been poorly investigated.

Therefore, the purpose of this study was to quantify the relationship between sedentary time and body composition in patients with T2D.

## Materials and methods

### Participants

We recruited T2D patients in the Endocrinology Department of the Second Affiliated Hospital of Nantong University from March 2020 to May 2021. The inclusion criteria were as follows: patients who: (1) met the 1999 World Health Organization (WHO) T2D diagnosis and classification criteria [19]; (2) were 25 to 75 years old; and (3) were fully aware of the purpose and significance of this study and were willing to sign up to participate. The exclusion criteria were as follows: (1) type 1 diabetes, gestational diabetes and other special types of diabetes; (2) a history of metabolic diseases or diseases affecting nutritional status, such as hyperthyroidism, hypothyroidism, Cushing syndrome, rheumatoid arthritis, etc.; (3) complication with severe chronic diseases, severe infections, immune disorders and malignant tumours; and (4) treatment with glucocorticoids or sex hormones in the past 6 months. Finally, we included 402 eligible patients with complete data for statistical analysis. The study was reviewed and approved by the Ethics Committee of the Second Affiliated Hospital of Nantong University and was in line with the Helsinki Declaration. In addition, all participants provided informed consent when recruited for the study.

### Data collection

A self-designed questionnaire was used to collect daily sedentary time, demographic data and medical history from all recruited patients. Patients were further divided into three subgroups: low sedentary time (LST, < 4 h), middle sedentary time (MST, 4–8 h) and high sedentary time (HST, > 8 h). Demographic data included age, sex, height, weight, waist circumference (WC), body mass index (BMI), systolic blood pressure (SBP), and diastolic blood pressure (DBP). Body mass index was calculated as follows: BMI = weight (kg)/height (m)^2^. Medical history included diabetes duration, history of hypertension, dietary habits (seafood, milk, soft drinks, tea, coffee, etc.), smoking and drinking history, and glucose-lowering therapies. Glucose-lowering therapies were categorized as insulin secretagogues, insulin, metformin, pioglitazone, α-glucosidase inhibitors (AGIs), dipeptidyl peptidase-4 inhibitors (DPP-4Is), sodium-glucose cotransporter-2 inhibitors (SGLT-2Is) and glucagon-like peptide-1 receptor agonists (GLP-1RAs).

For blood samples collection, each patient fasted overnight for 8 h, and the nurses in the ward took blood from the antecubital vein in the early morning hours of the next day for measurement of biochemical markers. Fasting glucose, triglycerides (TGs, colorimetric method), total cholesterol (TC, cholesterol oxidase method), low-density lipoprotein cholesterol (LDLC, selective melting method) and high-density lipoprotein cholesterol (HDLC, enzyme modification method) were measured by an automatic biochemical instrument (model 7600, Hitachi). The level of HbA1c was assessed by ion exchange high-performance liquid chromatography. The levels of fasting insulin, N-terminal osteocalcin (N-MID), β-collagen special sequence (β-CTX) and total type I procollagen N-terminal extension peptide (TP1NP), 25-hydroxyvitamin D (25(OH)D) and parathyroid hormone (PTH) were quantified by electrochemiluminescence immunoassay. All biochemical indices were assessed by professional doctors in the medical laboratory department of our hospital. Basal insulin resistance was assessed by homeostasis model assessment of insulin resistance (HOMA-IR), which was defined as follows: HOMA-IR = (fasting glucose × fasting insulin)/22.5. HOMA-IR was natural log-transformed for the further data analysis (lnHOMA-IR).

All the subjects in this study underwent scanning with a dual energy X-ray absorptiometry (DXA) (HologicDiscoveryWi, S/N86856) to assess the body composition of T2D patients, and scanning was performed by professionals in the corresponding medical and technical departments. All operations were carried out in accordance with the specifications of the instrument manual: the patient lay flat and was scanned from the head to the feet in the standard mode. The width of the scanning range was fixed at 60 cm, and the scanning time was approximately 20 min. The measured indices included lumbar L1–L4 bone mineral density (LS-BMD), femoral neck bone mineral density (FN-BMD), hip bone mineral density (H-BMD), total (whole-body) bone mineral density (T-BMD), whole-body fat mass (B-FAT), trunk fat mass (T-FAT) and limb skeletal muscle mass (ASMI). Bone mineral density (g/cm^2^) = bone mineral content (g)/area (cm^2^); ASMI evaluated by limb skeletal muscle mass = limb skeletal muscle mass (kg)/height^2^ (m^2^); and trunk fat percentage = trunk fat mass (g)/whole-body fat mass (g).

### Statistical analysis

Clinical variables of recruited patients are exhibited for all participants and the LST (< 4 h), MST (4–8 h) and HST (> 8 h) subgroups. Descriptive statistics for the data, including the mean with standard deviation and frequency with percentage, were analysed according to the data type. One-way analysis of variance (ANOVA) with linear polynomial contrasts and the chi-squared test with linear-by-linear association were performed to detect the trends of continuous data and categorical data in the three subgroups, respectively. In addition, we used GraphPad Prism to generate scatter plots of body composition indices in the LST (< 4 h), MST (4–8 h) and HST (> 8 h) subgroups.

Furthermore, we used multivariate linear regression analysis to adjust for other clinically relevant variables to detect the mean differences [B; 95% confidence interval (CI)] in body composition indices among the three subgroups with different sedentary times (LST, MST and HST), with the LST set as the reference value. The body composition indices included LS-BMD, FN-BMD, H-BMD, T-BMD, ASMI, B-FAT and T-FAT. Model 0 was unadjusted;  Model 1, Model 2 and Model 3 were gradually adjusted for other relevant clinical factors.

We used IBM SPSS Statistics (Version 22.0) and GraphPad Prism (Version 9.0) to analyse the data. Statistical significance was identified based on a threshold *p* value < 0.05.

## Results

### Clinical characteristics of patients

**Fig. 1:**
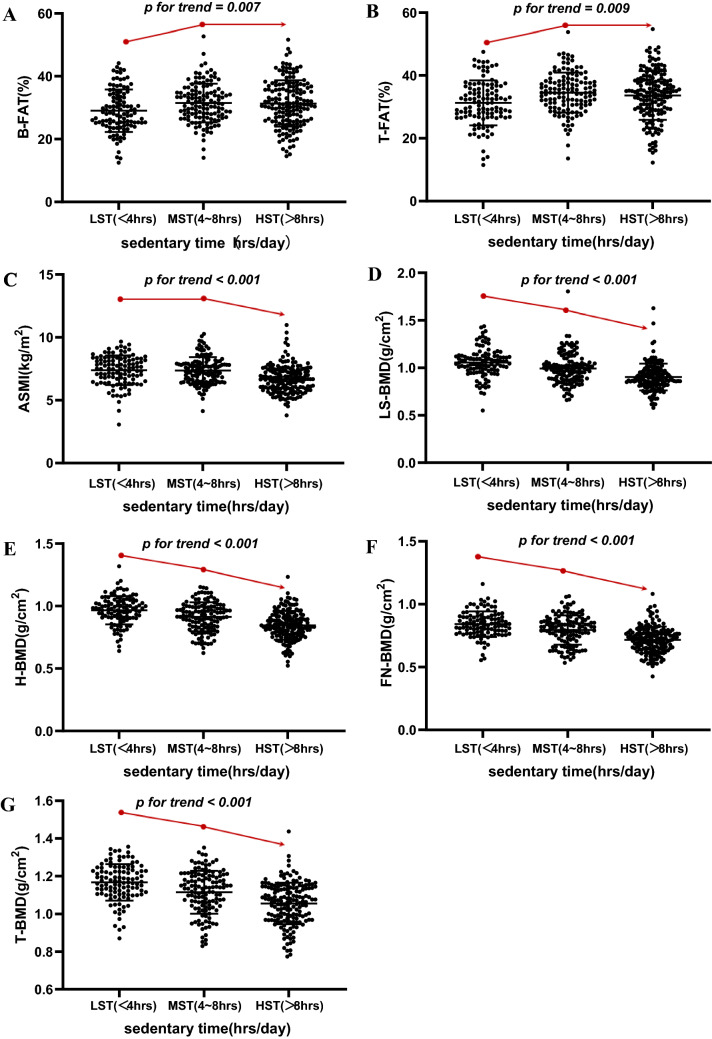
Scatter plots of body composition indices in three subgroups with low sedentary time (LST), middle sedentary time (MST) and high sedentary time (HST). **A** body fat pertencage (B-FAT); **B** trunk fat pertencage (T-FAT); **C** appendicular skeletal muscle index (ASMI); **D** lumbar spine bone mineral density (LS-BMD); **E** hip bone mineral density (H-BMD); **F** femoral neck bone mineral density (FN-BMD); **G** total bone mineral density (T-BMD)

Table 1 shows the basic clinical characteristics of 402 participants, who were divided into three groups according to the length of sedentary time: LST (< 4 h), MST (4–8 h), and HST (> 8 h). There were 109 (27.1%), 129 (32.1%) and 164 (40.8%) participants in each respective group. With the increase in sedentary time in T2D patients, the main parameters of body composition, such as LS-BMD, FN-BMD, H-BMD, T-BMD and ASMI, decreased significantly (*p* < 0.001). For the patients with sedentary time ≥ 4 h, B-FAT and T-FAT were higher than those in patients with sedentary time < 4 h (*p* = 0.009), which is shown in Fig. 1. In addition, with the increase in sedentary time, the duration of diabetes increased, HbA1c increased, the levels of N-MID and 25(OH)D decreased, and the levels of β-CTX, TP1NP and PTH increased (*p* < 0.001). Regarding the living habits of patients, as the duration of sedentary time increased, a higher frequency of drinking soft drinks, tea or coffee, smoking and drinking and a lower frequency of eating seafood and drinking milk were observed. However, there was no significant difference in sex, BMI, WC, HC, SBP, history of hypertension, TG, TC, HDLC or LDLC with prolonged sedentary time (*p* > 0.05). Among the glucose-lowering therapies, the frequency of AGIs, SGLT-2Is and GLP-1RAs use increased with the prolongation of sedentary time, while lifestyle alone and use of insulin , insulin-secretagogues, metformin, pioglitazone and DPP-4Is remained the same.

**Table 1:** Clinical characteristics of the total patients and subgroups based on the sedentary time

Variables	Total	Sedentary time			*F/x*^*2*^ *value*	*p for trend*
		LST (< 4 h)	MST (4–8 h)	HST (> 8 h)		
*n*	402	109(27.1)	129(32.1)	164(40.8)	–	–
Female, *n*(%)	173(43.0)	44(40.0)	58(45.4)	71(42.7)	0.170	0.680
Age(year)	55.20 ± 10.45	53.06 ± 9.11	55.21 ± 10.96	56.60 ± 10.71	7.611	0.006
BMI(kg/m^2^)	25.4 ± 3.7	25.2 ± 3.2	26.5 ± 3.6	24.8 ± 4.1	0.900	0.343
WC(cm)	90.5 ± 10.2	89.4 ± 8.4	93.2 ± 10.6	89.1 ± 10.5	0.053	0.891
SBP(mmHg)	133.0 ± 14.4	132.7 ± 14.2	134.7 ± 14.5	131.7 ± 14.3	0.287	0.593
DBP(mmHg)	81.6 ± 9.2	82.8 ± 8.7	82.7 ± 9.0	79.8 ± 9.4	6.873	0.009
Diabetes duration(year)	7.48 ± 5.70	5.26 ± 4.20	6.86 ± 5.41	9.44 ± 6.14	38.72	< 0.001
Glucose-lowering therapies						
Lifestyle alone, *n*(%)	33(8.2)	9(8.3)	16 (12.4)	8(4.9)	1.517	0.218
Insulin treatments, *n*(%)	183(45.5)	47(43.1)	50(38.8)	86(52.4)	2.954	0.086
Insulin-secretagogues, *n*(%)	98(24.4)	26(23.9)	33(25.6)	39(23.8)	0.003	0.954
Metformin, *n*(%)	175(43.5)	45(41.3)	63(48.8)	67(40.9)	0.058	0.810
Pioglitazone, *n*(%)	64(15.9)	18(16.5)	17(13.2)	5(17.7)	0.141	0.707
AGIs, *n*(%)	96(23.9)	16(14.7)	35(27.1)	45(27.4)	5.203	0.023
DPP-4Is, *n*(%)	53(13.2)	12(11.0)	17(13.2)	24(14.6)	0.74	0.390
SGLT-2Is, *n*(%)	97(15.2)	23(21.1)	29(22.5)	45(27.4)	5.199	0.023
GLP-1RAs, *n*(%)	46(11.4)	8(7.3)	10(7.8)	28(17.1)	6.966	0.008
Hypertension, *n*(%)	179(44.5)	44(40.4)	74(57.4)	61(37.2)	0.853	0.356
Smoking, *n*(%)	142(35.3)	25(22.9)	35(27.1)	82(50.0)	23.22	< 0.001
Alcohol consumption, *n*(%)	226(56.2)	44(40.4)	50(38.8)	132(80.5)	49.74	< 0.001
Seafood, *n*(%)	141(35.2)	49(45.0)	50(38.8)	42(25.8)	11.16	0.001
Milk, *n*(%)	149(37.1)	54(49.5)	46(35.7)	49(29.9)	10.38	0.001
Soft drink, *n*(%)	304(75.6)	68(62.4)	91(70.5)	145(88.4)	25.55	< 0.001
Tea or coffee, *n*(%)	209(52.3)	38(35.2)	51(39.5)	120(73.6)	43.06	< 0.001
TG(mmol/L)	2.51 ± 1.88	2.49 ± 1.65	2.99 ± 2.13	2.14 ± 1.73	2.436	0.119
TC(mmol/L)	4.36 ± 0.97	4.20 ± 0.97	4.42 ± 0.83	4.41 ± 1.05	3.191	0.075
HDLC(mmol/L)	1.15 ± 0.30	1.17 ± 0.28	1.12 ± 0.30	1.17 ± 0.30	0.016	0.900
LDLC(mmol/L)	2.82 ± 0.85	2.89 ± 0.83	2.74 ± 0.81	2.84 ± 0.89	0.206	0.650
N-MID(ng/mL)	11.7 ± 3.8	12.43 ± 4.03	11.78 ± 3.50	11.16 ± 3.83	7.372	0.007
β-CTX(ng/mL)	0.46 ± 0.20	0.43 ± 0.20	0.43 ± 0.18	0.49 ± 0.21	5.538	0.019
TP1NP(ng/mL)	40.22 ± 12.33	38.21 ± 11.59	39.34 ± 11.40	42.24 ± 13.24	7.058	0.008
25(OH)D(ng/mL)	17.5 ± 6.5	18.83 ± 6.11	18.12 ± 6.95	16.12 ± 6.11	11.76	0.001
PTH(pg/mL)	40.70 ± 16.91	35.86 ± 13.45	41.81 ± 20.10	43.03 ± 15.64	12.10	0.001
HbA1c(%)	9.04 ± 1.76	8.53 ± 1.68	8.89 ± 1.52	9.50 ± 1.87	21.00	< 0.001
HOMA-IR	3.29 ± 3.52	3.22 ± 4.63	3.55 ± 3.05	3.12 ± 2.99	0.047	0.828
lnHOMA-IR	0.81 ± 1.01	0.69 ± 1.08	0.91 ± 1.00	0.82 ± 0.96	0.952	0.330
B-FAT(%)	30.76 ± 6.89	29.04 ± 6.75	31.48 ± 6.13	31.33 ± 7.38	7.357	0.007
T-FAT (%)	33.28 ± 7.27	31.29 ± 7.15	34.54 ± 6.37	33.63 ± 7.76	6.949	0.009
ASMI(kg/m^2^)	7.09 ± 1.16	7.38 ± 1.18	7.36 ± 1.07	6.68 ± 1.11	25.81	< 0.001
LS-BMD(g/cm^2^)	0.97 ± 0.16	1.05 ± 0.15	0.99 ± 0.16	0.90 ± 0.14	66.35	< 0.001
FN-BMD(g/cm^2^)	0.78 ± 0.12	0.84 ± 0.10	0.79 ± 0.12	0.72 ± 0.11	86.43	< 0.001
H-BMD(g/cm^2^)	0.90 ± 0.12	0.97 ± 0.11	0.91 ± 0.17	0.85 ± 0.11	71.65	< 0.001
T-BMD(g/cm^2^)	1.10 ± 0.12	1.17 ± 0.10	1.12 ± 0.11	1.06 ± 0.11	70.39	< 0.001

ANOVA followed by a post-test for linear trend and linear-by-linear association chi-squared test were applied to detect trends in continuous data and categorical data among sedentary time, respectively. Corresponding test statistics and *p* values for trends are also provided

### Mean differences in body composition parameters between the three sedentary duration groups

Tables 2 and 3 present multivariate linear regression models, taking the LST (< 4 h) group as a reference, to display the mean changes of body composition parameters in the MST (4–8 h) and HST (> 8 h) groups (B[95%CI]). The mean differences in B-FAT, T-FAT, ASMI, LS-BMD, FN-BMD, H-BMD and T-BMD between the LST (< 4 h) group and HST (> 8 h) group were 2.289 (0.630 to 3.948)%, 2.338 (0.594 to 4.082)%, − 0.700(− 0.971 to − 0.429) kg/m^2^, − 0.152(− 0.189 to − 0.115) g/cm^2^, − 0.125(− 0.152 to − 0.099) g/cm^2^, − 0.121(− 0.149 to − 0.093) g/cm^2^ and − 0.112(− 0.138 to − 0.086) g/cm^2^, respectively. After adjustment for other relevant clinical factors and with the LST group as the reference, the adjusted mean changes [B (95% CI)] in B-FAT, T-FAT, ASMI, LS-BMD, FN-BMD, H-BMD and T-BMD in the HST group were 2.011(1.014 to 3.008)%, 1.951(0.705 to 3.197)%, − 0.377(− 0.531 to − 0.223) kg/m^2^, − 0.083(− 0.124 to − 0.042) g/cm^2^, − 0.051(− 0.079 to − 0.024) g/cm^2^, − 0.059(− 0.087 to − 0.031) g/cm^2^ and − 0.060(− 0.088 to − 0.033) g/cm^2^, respectively. Sedentary time was independently and positively correlated with B-FAT and T-FAT, and was independently and negatively correlated with ASMI, LS-BMD, FN-BMD and H-BMD and T-BMD in T2D patients.

**Table 2:** Mean differences of muscle mass and fat distribution between the subgroups with different sedentary time [B (95%CI)]

Models	Sedentary time			*t value*	*p for trend*	Adjusted *R*^*2*^
	LST (< 4 h)	MST (4–8 h)	HST (> 8 h)			
ASMI						
Model 0	0-reference	− 0.020(− 0.305 to 0.265)	− 0.700(− 0.971 to − 0.429)	− 5.391	< 0.001	0.060
Model 1	0-reference	0.042(− 0.185 to 0.270)	− 0.660(− 0.876 to − 0.444)	− 6.395	< 0.001	0.395
Model 2	0-reference	− 0.057(− 0.224 to 0.110)	− 0.331(− 0.510 to − 0.152)	− 3.671	< 0.001	0.715
Model 3	0-reference	− 0.094(− 0.237 to 0.050)	− 0.377(− 0.531 to − 0.223)	− 4.865	< 0.001	0.799
B-FAT						
Model 0	0-reference	2.439(0.693 to 4.186)	2.289(0.630 to 3.948)	2.519	0.012	0.013
Model 1	0-reference	1.994(0.754 to 3.234)	2.005(0.827 to 3.183)	3.137	0.002	0.502
Model 2	0-reference	0.663(− 0.314 to 1.640)	2.005(0.959 to 3.050)	3.790	< 0.001	0.723
Model 3	0-reference	0.560(− 0.369 to 1.490)	2.011(1.014 to 3.008)	4.011	< 0.001	0.760
T-FAT						
Model 0	0-reference	3.249(1.407 to 5.091)	2.338(0.594 to 4.082)	2.325	0.021	0.011
Model 1	0-reference	2.933(1.437 to 4.429)	2.090(0.675 to 3.506)	2.548	0.011	0.345
Model 2	0-reference	1.327(0.120 to 2.534)	2.050(0.762 to 3.337)	3.120	0.002	0.624
Model 3	0-reference	1.047(− 0.118 to 2.213)	1.951(0.705 to 3.197)	3.079	0.002	0.667

Model 0: unadjusted

Model 1: additionally adjusted for sex

Model 2: additionally adjusted for age, WC, SBP, DBP, diabetes duration, hypertension, smoking, alcohol and glucose-lowering therapies

Model 3: additionally adjusted for BMI, HbA1c, lnHOMA-IR and lipid profiles

**Table 3:** Mean differences of bone mineral density between the subgroups with different sedentary time (B [95%CI])

Model	Sedentary time			*t value*	*p for trend*	Adjusted *R*^*2*^	
	LST (< 4 h)	MST (4–8 h)	HST (> 8 h)				
LS-BMD							
Model 0	0-reference	− 0.063(− 0.102 to − 0.025)	− 0.152(− 0.189 to − 0.115)	− 8.301	< 0.001	0.145	
Model 1	0-reference	− 0.070(− 0.108 to − 0.032)	− 0.153(− 0.190 to − 0.115)	− 8.154	< 0.001	0.225	
Model 2	0-reference	− 0.047(− 0.084 to − 0.010)	− 0.098(− 0.138 to − 0.058)	− 4.841	< 0.001	0.318	
Model 3	0-reference	− 0.043(− 0.080 to − 0.006)	− 0.083(− 0.124 to − 0.042)	− 3.957	< 0.001	0.347	
FN-BMD							
Model 0	0-reference	− 0.046(− 0.074 to − 0.018)	− 0.125(− 0.152 to − 0.099)	− 9.517	< 0.001	0.184	
Model 1	0-reference	− 0.045(− 0.070 to − 0.020)	− 0.109(− 0.133 to − 0.084)	− 8.941	< 0.001	0.399	
Model 2	0-reference	− 0.027(− 0.051 to − 0.003)	− 0.065(− 0.092 to − 0.039)	− 4.934	< 0.001	0.473	
Model 3	0-reference	− 0.022(− 0.047 to 0.002)	− 0.051(− 0.079 to − 0.024)	− 3.759	< 0.001	0.493	
H-BMD							
Model 0	0-reference	− 0.053(− 0.082 to − 0.023)	− 0.121(− 0.149 to − 0.093)	− 8.065	< 0.001	0.156	
Model 1	0-reference	− 0.057(− 0.082 to − 0.032)	− 0.102(− 0.127 to − 0.077)	− 8.045	< 0.001	0.418	
Model 2	0-reference	− 0.042(− 0.067 to − 0.017)	− 0.069(− 0.096 to − 0.042)	− 4.968	< 0.001	0.479	
Model 3	0-reference	− 0.039(− 0.064 to − 0.014)	− 0.059(− 0.087 to − 0.031)	− 4.071	< 0.001	0.493	
T-BMD							
Model 0	0-reference	− 0.052(− 0.080 to − 0.024)	− 0.112(− 0.138 to − 0.086)	− 8.496	< 0.001	0.151	
Model 1	0-reference	− 0.047(− 0.071 to − 0.022)	− 0.102(− 0.126 to − 0.078)	− 8.434	< 0.001	0.369	
Model 2	0-reference	− 0.033(− 0.057 to − 0.009)	− 0.068(− 0.094 to − 0.042)	− 5.122	< 0.001	0.439	
Model 3	0-reference	− 0.031(− 0.056 to − 0.007)	− 0.060(− 0.088 to − 0.033)	− 4.365	< 0.001	0.446	

Model 0: unadjusted

Model 1: adjusted for age, sex, WC, SBP, DBP, BMI and diabetes duration

Model 2: additionally adjusted for hypertension, seafood, milk, soft drink, tea or coffee, smoking, alcohol and glucose-lowering therapies

Model 3: additionally adjusted for HbA1c, lnHOMA-IR, lipid profiles, N-MID, β-CTX, TP1NP, 25(OH)D and PTH

## Discussion

In this study, we systematically analysed the relationship between sedentary time and body composition in 402 patients with T2D. First, through univariate analysis, sedentary time was found to be closely related to B-FAT, T-FAT, ASMI, LS-BMD, FN-BMD, H-BMD and T-BMD in T2D patients; second, multiple linear regression showed that sedentary duration was independently correlated with B-FAT, T-FAT, ASMI, LS-BMD, FN-BMD and H-BMD and T-BMD in T2D patients. Compared with the LST group, the HST group showed an increase in B-FAT and T-FAT, and the corresponding adjusted mean changes were 2.011(1.014 to 3.008) and 1.951(0.705 to 3.197), respectively. Additionally, compared with the LST group, HST group exhibited a decrease in ASMI, LS-BMD, FN-BMD, H-BMD and T-BMD, and the corresponding adjusted mean changes were − 0.377(− 0.531 to − 0.223), − 0.083(− 0.124 to − 0.042), − 0.051(− 0.079 to − 0.024), − 0.059(− 0.087 to − 0.031), and − 0.060(− 0.088 to − 0.033), respectively. Third, with the increase in sedentary time in T2D patients, HbA1c levels were significantly increased, while there were no significant differences in TG, TC and HDLC levels between the three subgroups with different sedentary behaviors.

Studies have shown that the biological and metabolic characteristics of fat and muscle tissue in different parts of the body are different. In general, the risk of metabolic diseases is proportional to the total amount of fat in the body. Abdominal obesity caused by the accumulation of abdominal visceral adipose tissue (VAT) is more closely related to hypertension, dyslipidaemia and insulin resistance (IR) than obesity caused by the accumulation of adipose tissue around the lower extremities and buttocks [20, 21]. The study found that 72% of T2D patients have increased abdominal fat deposition and that abdominal obesity may be an independent risk factor for metabolic syndrome components such as diabetes and lipid metabolic disorders [22, 23]. In this study, the average BMI of the three sedentary duration groups was ≥ 24 kg/m^2^ [24]. Choi et al*.* [7] found that trunk and arm fat were higher in T2D patients than in non-diabetes people and that every 1 kg increase in body fat increased the incidence of diabetes by 15% in men and 19% in women. The same result was found for upper limb fat after multifactor adjustment. In contrast, with each 1 kg increase in lower limb fat, the prevalence of diabetes decreased by 51% in men and 44% in women. The difference in metabolism of different types of adipose tissue is related to their histological and biological differences. Compared with subcutaneous adipose tissue (SAT), abdominal VAT is composed of larger adipocytes that secrete more IR-related molecules [25]. VAT has a more abundant vascular system and nerve distribution and has strong metabolic activity. Its decomposition produces a large number of free fatty acids, adipocytokines and inflammatory factors, which promote glycogen heterogeny, lipid synthesis and IR enhancement, leading to the occurrence of metabolic diseases such as glucose and lipid metabolism disorders [25]. Studies have shown that lack of exercise and prolonged sitting are the main factors leading to obesity and IR [26, 27].

Bones and muscles make up the skeletal muscle system. An increasing number of studies have shown that bones and muscles share common paracrine and endocrine regulation. In ageing and chronic diseases, bone loss and muscle atrophy occur at the same time [28]. Muscle mass peaks around the age of 25, followed by loss of muscle mass after the age of 30, a slight decrease in the number of muscle fibers at the age of 50 and rapid decline after the age of 60. Similar to muscle loss, bone mass increases before age 30, peaks around age 30, and then enters a phase of bone loss, which is more pronounced in postmenopausal women. Although bone loss and muscle atrophy were corrected with aging, appendicular skeletal muscle mass(ASMI) and BMD of the hip, lumbar spine, femoral neck and total body were decreased with the increasing of sedentary time (p for trend < 0.01), even after adjusting for age. Studies have shown that an increase or decrease in muscle mass is positively correlated with an increase or decrease in BMD, respectively, and that the loss of skeletal muscle can lead to a decrease in BMD. Muscle atrophy, muscle strength decline and muscle dysfunction can accelerate thin cortical bone absorption, weaken the ability to resist shear force, torsion and bending force, reduce the number of horizontal trabeculae in cancellous bone, diminish the vertical trabeculae, and decrease BMD [29]. In addition, there has been growing clinical concern with the effect of glucose-lowering agents on bone metabolism. In our study, we found the frequency of AGIs, SGLT-2Is and GLP-1RAs use increased with the prolongation of sedentary time. These glucose-lowering therapies were more frequent in the T2D patients with longer sedentary time and higher HbA1c, and may have effects on bone mass and bone mineral density in these T2D patients. A previous study has shown that liraglutide, one of the GLP-1RAs, can improve bone mineral density and bone quality while reducing blood glucose in diabetic rats [30]. SGLT-2Is can increase the reabsorption of phosphorus in renal tubules, which may affect calcium and phosphorus metabolism, increase blood phosphorus, stimulate PTH secretion and enhance bone resorption. In a multicenter RCT study, 104-week treatment with canagliflozin may result in a mild but significant decrease in bone mineral density of the total hip rather than the femoral neck, lumbar vertebrae or distal forearm in women with T2D when compared to the placebo group [31]. However, fewer studies are available in the field of AGIs with bone metabolism [32]. In our present study, we should consider the effects of glucose-lowering therapies on bone metabolism. Furthermore, after adjusted for these glucose-lowering therapies by multivariate linear regression analysis, we found that different sedentary time was responsible for body composition independent of glucose-lowering therapies.

The changes in human muscle mass are related to poor nutrition, the weakening of hormones, metabolism, and immune function, and the degeneration of motor units and muscle fibres [33]. Muscle fibre degeneration leads to decreased muscle mass, and muscle fibre atrophy is one of the causes of sarcopenia. In T2D patients, IR, ectopic lipid deposition, oxidative stress, endoplasmic reticulum stress, accumulation of advanced glycation end-products (AGEs), neuropathy and nephropathy can all cause damage. The synthesis and decomposition balance of skeletal muscle [34, 35] and IR are the main pathogenic mechanisms of sarcopenia caused by diabetes. IR reduces protein synthesis by inhibiting the activation of the skeletal muscle differentiation signalling pathway and increases muscle protein decomposition by activating the ubiquitin–proteasome pathway [36]. AGE accumulation can inhibit the expression of myogenic genes and osteocalcin in myoblasts, thus aggravating muscle and bone loss [37]. Studies have shown that approximately 50% of diabetes patients have diabetic osteoporosis (OP), and with the extension of the course of diabetes, the incidence of diabetic OP gradually increases. Second, a large number of epidemiological studies have shown that the risk of hip, foot and proximal femoral fracture in patients with diabetes is significantly higher than that in nondiabetic people. Tebé et al*.* [38] conducted a follow-up study of 50,000 T2D patients and 100,000 nondiabetic people for 6 to 8 years and showed that after excluding the confounding factor of death, the risk of hip fracture in T2D patients was still higher than that of nondiabetic people. In T2D patients, insulin deficiency leads to a decrease in renal tubular reabsorption, loss of calcium and phosphorus, bone calcium mobilization, increase in bone resorption, decrease in bone formation and decrease in BMD. At the same time, insulin deficiency inhibits osteocalcin synthesis by osteoblasts, thus weakening bone mineralization and bone cell activity and inhibiting bone formation [39]. OP increases the risk of falls, while reduced muscle mass and decreased function reduce strength, lead to limited movement, and increase the risk of falls and fractures [40].

There is evidence that sedentary behaviour has a direct effect on metabolism, bone mineral content and vascular health [41]. Excessive sedentary behaviour can aggravate chronic diseases. One of the effects of sedentary behaviour is metabolic disorder, which is characterized by an increase in TG levels, a decrease in HDLC levels, a reversible decrease in multiple organ insulin sensitivity and cardiopulmonary adaptability, and an increase in liver fat and dyslipidaemia, resulting in metabolic disorders and changes in body composition [42]. This is because prolonged sedentary time may lead to a decrease in skeletal muscle contractile activity, thereby reducing the activity of lipoprotein lipase (LPL) in muscle [43]. In addition, rat experiments have shown that there is a relationship between LPL and limb activity [44]. On the one hand, the sedentary state decreases the physiological activity of muscle, which leads to a decrease in LPL activity and oxidation disorder, which has an adverse effect on muscle fibres. On the other hand, sedentary behaviour reduces the basal metabolism of the body, which is often accompanied by an increase in food intake and a decrease in energy output, resulting in weight gain and an increase in glucose concentration, which further promotes the occurrence of T2D [45]. Therefore, an increase in physical activity can induce an increase in LPL activity and have a positive effect on metabolic glycosylated fibres [46]. However, in our study, the TG levels in the HST group had decreased trend than MST group. In addition, the HDLC level did not change between these three groups. T2D patients in our study have received glucose-lowering therapies. Glucose-lowering agents, such as SGLT-2Is and GLP-1RAs, can regulate serum lipid metabolism [47–49], which may account for the differences of lipid profiles between the present and previous studies. At the same time, interrupting sitting for a long time through simple activities such as standing or walking can reduce the concentration of postprandial glucose and insulin [50, 51], which plays an important role in the prevention and control of T2D. Another adverse effect of sedentary behaviour is a decrease in BMD. The relationship between sedentary behaviour and osteopenia is regulated by changes in the balance between bone resorption and deposition. Sedentary behaviour will lead to a rapid increase in bone resorption but will not be accompanied by changes in bone formation, resulting in a decrease in bone mineral content and an increase in the risk of OP. In addition, sedentary time is related to decreased body function and blood flow in the legs, which may lead to individual falls, bone loss and fractures. LaMonte et al*.* [52] found that regular physical activity and less sedentary time reduced the risk of fracture in older women and that women who spent more than 9.5 h a day being sedentary had a 4% increased risk of fracture compared with those who had the shortest sedentary time. A meta-analysis evaluated the relationship between total sedentary time and all-cause mortality [11]. Another meta-analysis reported that adults with a sedentary time of more than 8 h a day have a higher risk of death from cardiovascular disease, while adults with a higher exercise level have a lower risk of death [12]. At the same time, there is a nonlinear dose–response relationship between sedentary time and all-cause mortality, cardiovascular mortality, cancer mortality and cardiovascular adverse events, but it varies with exercise levels [12, 53]. In general, sedentary behaviour had a stronger impact on health for people with a low exercise level but had less impact on people who performed moderate- or even high-intensity exercise for a long time [53]. HUNT Study explored the interaction between sedentary time and physical activity and found that, compared with people who were sedentary for less than 4 h a day, people with a daily sedentary time of 5–7 h and those with more than 8 h had a 26% and 30% increased risk of T2D, respectively [54]. In China, the average daily sedentary time of diabetes patients is as high as 6.1 h. The sedentary time of T2D patients can account for 70% of their daily waking time. To reduce the harmful effects of sedentary behaviour on health, sedentary time should be shortened as much as possible. For people who cannot avoid being sedentary due to the nature of their work, exercise at higher than the recommended level should be carried out every week to mediate the health hazards of being sedentary to a certain extent.

## Limitations

First, the subjects selected in this study included men and women in a large age range (25–75 years old). T2D patients of a single sex and in the same age group have not been studied, but this study is closer to the clinical situation. Second, this study is only a cross-sectional study and did not perform a follow-up survey. The results of the study cannot be compared before and after, and a follow-up survey is needed to confirm the authenticity and reliability of the research results.

## Conclusions

A sedentary lifestyle may account for increases in trunk and body fat percentage and decreases in appendicular skeletal muscle mass and BMD of the lumbar spine, femoral neck, hip and total body in patients with T2D. These results may indicate that sedentary time may have an effect on body composition in T2D.

## Data Availability

The study data could be provided to the interested researchers upon reasonable requests. The requests for data should be made to the corresponding author of the study.

## Abbreviations

T2D: Type 2 diabetes
LST: Low sedentary time
MST: Middle sedentary time
HST: High sedentary time
DXA: Dual energy X-ray absorptiometry
B-FAT: Body fat percentage
T-FAT: Trunk fat percentage
ASMI: Appendicular skeletal muscle index
BMD: Bone mineral density
LS-BMD: Lumbar spine BMD
FN-BMD: Femoral neck BMD
H-BMD: Hip BMD
T-BMD: Total BMD
WC: Waist circumference
SBP/DBP: Systolic/diastolic blood pressure
BMI: Body mass index
AGIs: α-Glucosidase inhibitors
DPP-4Is: Dipeptidyl peptidase-4 inhibitors
SGLT-2Is: Sodium-glucose cotransporter-2 inhibitors
ANOVA: One-way analysis of variance
TG: Triglycerides
TC: Total cholesterol
HDLC: High-density lipoprotein cholesterol
LDLC: Low-density lipoprotein cholesterol
HbA1c: Glycosylated hemoglobin A1c
IR: Insulin resistance
HOMA-IR: Homeostasis model assessment of insulin resistance
lnHOMA-IR: Natural log-transformed HOMA-IR
N-MID: N-terminal osteocalcin
β-CTX: β-Collagen special sequence
TP1NP: Total type I procollagen N-terminal extension peptide
25(OH)D: 25-Hydroxyvitamin D
PTH: Parathyroid hormone
OP: Osteoporosis
